# Strategies to reduce road traffic injuries among motorcyclists in Dezful, Iran: stressing on legal and environmental factors

**DOI:** 10.5249/jivr.v14i1.1696

**Published:** 2022-01

**Authors:** Maryam Mazaheri, Majid Rezai-Rad, Ferdos Pelarak

**Affiliations:** ^*a*^ School of Medicine, Dezful University of Medical Sciences (DUMS), Dezful, Iran.; ^*b*^ Department of Healthcare Service Management, School of Management, Islamic Azad University, South Tehran Branch, Tehran, Iran.; ^*c*^ Department of Nursing and Midwifery, Islamic Azad University, Dezful Branch, Dezful, Iran.

**Keywords:** Traffic accidents, Motorcycles, Road traffic injuries (RTIs), Environmental factors, Legislation

## Abstract

**Background:**

Road traffic injuries (RTIs) have been eighth leading cause of death in the world and second leading one Iran in 2018. Every year, a large number of motorcycle RTIs lead to deaths and disabilities due to non-compliance with traffic rules and the traditional design of the streets and routes in Dezful, Iran. This study aims to pursue two goals: identifying the legal and environmental factors affecting motorcycle RTIs, and prioritizing effective strategies in reducing number of motorcycle RTIs in Dezful, Iran.

**Methods:**

A mixed method approach was used in this study. In the qualitative phase, focus group meetings using key informants were used to identify the effective factors and in the quantitative one a matrix was used for prioritizing effective strategies in preventing motorcycle RTIs.

**Results:**

45 basic codes related to legal factors and 8 basic codes of environmental factors were de-rived from the focus group meetings. Six main legal factors and 3 main environmental factors were prioritized as the most effective strategies to reduce motorcycle RTIs. The legal factors with the highest score were: making visible: obstacles, motorcycles and pedestrians and motor-cyclists using colors and stickers or glossy stickers, further monitoring and training of riders’ license issuance schools, seriousness in enforcing the laws and dealing legally and seriously with violators, continuous marking of roads and streets, random check of motorcycle riders' license, and construction of public parking lots in crowded zones. The environmental ones were: identifying places where traffic signs are covered with trees, and reporting through the 137 call center, identifying and reporting shoulderless and hazardous roads by municipality, and Identifying and reporting accident-causing potholes through the 137 call center.

**Conclusions:**

All organizations and stakeholders involved in reducing motorcycle RTIs, should take benefit from different recommendation - i.e. education & awareness, law enforcement and legal actions, environmental actions, collaborations, partnerships, and lobbying, and research.

## Introduction

Road traffic injuries (RTIs), has been the world's eighth leading cause of death. According to the WHO in 2018, RTIs have been the leading cause of death in the age group of 15-29 year. Studies indicate that globally, a RTI occurs every 30 seconds^1^ resulting in 1.24 million deaths, 10 million disabilities, and about 50 million injuries.^2^ Traffic crashes are unfortunate events that lead to unpredictable traumatic which can cause injuries.^3^ By 2030, it is projected that traffic fatalities will be the fifth leading cause of death and disability.^4^

***Road safety in the Asia-Pacific region***

Road safety is among the critical challenges for materialization of sustainable development in the Asia-Pacific region. Every 38 seconds, one person dies on the roads of the region due to not complying with traffic regulations. According to the 2018 WHO Global Status Report on Road Safety over 60% of the global road fatalities occurred in this region. The number of regional road fatalities grew by 3.5% on an average per year in 2013-2016. In 2016, the road traffic fatality rate in the region stood at 18.35 per 100,000 population- slightly higher than the global average fatality rate of 18.14. Over 97% of these fatalities occurred in the low-income and middle-income countries. In the Asia-Pacific region, vulnerable road users including 2 and 3 wheeler motorcyclists, pedestrians, and bicyclists accounted for 54.76% of the whole road fatalities.^5,6^

RTIs in Iran were the second leading cause of death after cardiovascular factors in 2018.^7^ It is estimated that more than 20-80 thousand deaths and injuries occur annually in Iran due to road traffic accidents.^3^ A WHO report, mentions that the death rate in Iran due to RTIs stood at 1.32%.^8^ If proper measures are not taken to reduce rate of traffic crashes, by 2030 it may result in about 2.4 million deaths and long-term physical and mental disabilities worldwide.^9^

A review of recent reports by the WHO shows that due to the increase in the number of vehicles over recent years, the death rate has remained somewhat stable. Of course, the rate is slightly higher in Iran and the city of Dezful. Examination of available statistics also shows that number of deaths due to traffic accidents has decreased, but the number of injuries are increasing.^5^

RTIs information is obtained in different ways in different cities of Iran. Sharifian et al. in their qualitative study to explain road traffic data collection and registry in Iran, found that due to various organizations at pre-incident and post-incident levels, there is a strong lack of integrated systems that can handle a large amount of information generated in these organizations. The following factors were identified as interventional factors in forming separated registration: lack of coherent registry infrastructure, lack of clarity of recording information, and lack of required resources for registration in this study. According to the finding of this study, some organizations in Iran do not have the technical infrastructure necessary for timely registration of information.^10^

***RTIs Effective factors***

The phenomenon of RTIs is a multifactorial problem, and adopting practical solutions to reduce accident' rates and prevention is essential.

According to the WHO reports, the factors effective in the occurrence of traffic accidents may be referred to as follows; of course, these factors do not have clear boundaries and overlap:^11^

• Human factors (drivers' physical and mental condition and their behavior),

• Environmental factors (roads construction and maintenance, visibility, roads slipperiness, climate changes, etc.),

• Legal factors (constituting regulation and law enforcement by the police),

• Vehicle-related factors (braking, steering and repairs, design defects, improving safety standards), and

• Managerial-organizational factors (evaluation of infrastructures, financial and human resources in law enforcement and constituting national policies).

In several studies, factors such as violation of laws, using cell phone, pedestrians' uncontrolled crossing on the roads^12^ weekends and nights, alcohol and drug use, speeding, climate conditions, lighting conditions, vehicle type, road conditions, type of collision and accident location^13^ including not been familiar with legal laws, and not enforcing traffic laws^14^ identified as contributing to RTIs. Suggestions to address RTIs included increasing penalties for driving while being intoxicated (DWI), institutionalizing the culture of using seat belts, setting up more speed cameras, traffic safety training for drivers in adverse weather conditions, providing adequate lighting on roads and streets, and designing safer vehicles.^15^

***Dezful: the city under study***

Dezful city is located in the north of Khuzestan province, southwest of Iran. Due to its geographical location and narrow streets, especially in the city center and for cultural reasons, many people ride motorbikes. Every year, according to the traffic police and studies conducted on the issue^16^ a large number of motorcycle RTIs lead to deaths and disabilities due to non-compliance with traffic rules and the traditional design of the streets and routes in the city. 

Insufficient research has been conducted on the related to motorcycle RTIs, including the identification and implementation of effective strategies to reduce rate of crashes in this city. For several reasons the present study has been conducted by a mixed method:

• Majority of conducted studies are in the field of human factors and less attention has been paid to other factors mentioned by WHO;

• There was no valid information at the national and city level on the factors contributing to RTIs;

• There were shortcomings in the studies conducted on legal and environmental factors;

• Most of the suggested solutions were related to the human factor and the driver (victim blaming);

• Legal (police and law enforcement) and environmental (road and weather) solutions were not enough dealt with in the interventions;

• Appropriate and practical data need to be provided for planning and designing future interventions to reduce RTIs.

Given the large number of studies conducted on human factors and the lack of enough studies conducted on environmental and legal factors, it was decided to conduct a study on both environmental and legal factors. The present study pursued two goals: 1) to identify the legal and environmental factors affecting RTIs among motorcyclists; 2) to prioritize effective strategies in reducing number of RTIs among motorcyclists.

## Methods 

***Research type and setting***

This study was conducted in Dezful by Dezful University of Medical Sciences (DUMS). In general, the weather in this city is warm and it is one of the tropical regions of the country that has dry winters and hot summers. The central parts of the city have an old structure, and in terms of expanding its structure are faced with some restrictions.

According to the reports of local officials, as well as the statistics provided by DUMS, everyday numerous traffic accidents occur in Dezful^5,6,16^ and of course motorcyclists play a prominent role in these accidents. It could be argued that the reasons for this problem could be attributed to the hot weather and nervousness of drivers, as well as the narrow streets in the old structures of the city, i.e. the main streets of the city where all businesses are concentrated. Hence, in addition to driving cars to handle their daily affairs many families have motorcycles so that they could pass through these narrow and busy streets easily and freely and manage their daily activities. Whereas the city is one of the agricultural and labor hubs of the country, many people use motorcycles due to its low cost and enabling the family members to handle their activities more easily. Therefore, the number of motorcycles and motorcyclists in this city is very high, and unfortunately, as a result accidents and deaths due to motorcycling as well as high-risk behaviors and violations are also high.

In addition to all these issues, according to the main researchers' field observations, who are residents of Dezful, unfortunately, no serious and strict legal action is taken to cope with these problems, and traffic police officers easily ignore the risky behaviors of motorcyclists.

The present research was in continuation with the previous phase of the motorcyclists' accident reduction program in Dezful (phase 1 was conducted with emphasis on human factors in 2015),^16^ from June to August 2017 using a mixed method approach with emphasis on environmental and legal factors. For standard reporting qualitative research findings, Standard Reporting Qualitative Research (SRQR) checklist was used, which is attached to the article as.appendix 1.

**appendix 1:** Standards For Reporting Qualitative Research (SRQR)*

NO.	TOPIC	ITEM	REPORTED ON PAGE #
		**TITLE AND ABSTRACT**	
S1	Title	Concise description of the nature and topic of the study Identifying the study as qualitative or indicating the approach (e.g., ethnography, grounded theory) or data collection methods (e.g., interview, focus group) is recommended	89
S2	Abstract	Summary of key elements of the study using the abstract format of the intended publication; typically includes background, purpose, methods, results, and conclusions	89
		**INTRODUCTION**	
S3	Problem formulation	Description and significance of the problem/phenomenon studied; review of relevant theory and empirical work; problem statement	89-91
S4	Purpose or research question	Purpose of the study and specific objectives or questions	91
		**METHODS**	
S5	Qualitative approach and research paradigm	Qualitative approach (e.g., ethnography, grounded theory, case study, phenomenology, narrative research) and guiding theory if appropriate; identifying the research paradigm (e.g., postpositivist, constructivist/ interpretivist) is also recommended; rationale	91-92
S6	Researcher characteristics and reflexivity	Researchers’ characteristics that may influence the research, including personal attributes, qualifications/experience, relationship with participants, assumptions, and/or presuppositions; potential or actual interaction between researchers’ characteristics and the research questions, approach, methods, results, and/or transferability	91-93
S7	Context	Setting/site and salient contextual factors; rationale	91-92
S8	Sampling strategy	How and why research participants, documents, or events were selected; criteria for deciding when no further sampling was necessary (e.g., sampling saturation); rationale	92,93,94
S9	Ethical issues pertaining to human subjects	Documentation of approval by an appropriate ethics review board and participant consent, or explanation for lack thereof; other confidentiality and data security issues	95,105
S10	Data collection methods	Types of data collected; details of data collection procedures including (as appropriate) start and stop dates of data collection and analysis, iterative process, triangulation of sources/methods, and modification of procedures in response to evolving study findings; rationale	92-93, 94-95
S11	Data collection instruments and technologies	Description of instruments (e.g., interview guides, questionnaires) and devices (e.g., audio recorders) used for data collection; if/how the instrument(s) changed over the course of the study	92-93, 94-95
S12	Units of study	Number and relevant characteristics of participants, documents, or events included in the study; level of participation (could be reported in results)	92, 95
S13	Data processing	Methods for processing data prior to and during analysis, including transcription, data entry, data management and security, verification of data integrity, data coding, and anonymization/de-identification of excerpts	93-94, 95
S14	Data analysis	Process by which inferences, themes, etc., were identified and developed, including the researchers involved in data analysis; usually references a specific paradigm or approach; rationale	93-95, 95
S15	Techniques to enhance trustworthiness	Techniques to enhance trustworthiness and credibility of data analysis (e.g., member checking, audit trail, triangulation); rationale	94
		**RESULTS/FINDINGS**	
S16	Synthesis and interpretation	Main findings (e.g., interpretations, inferences, and themes); might include development of a theory or model, or integration with prior research or theory	95-100
S17	Links to empirical data	Evidence (e.g., quotes, field notes, text excerpts, photographs) to substantiate analytic findings	96-98
		**DISCUSSION**	
S18	Integration with prior work, implications, transferability, and contribution(s) to the field	Short summary of main findings; explanation of how findings and conclusions connect to, support, elaborate on, or challenge conclusions of earlier scholarship; discussion of scope of application/generalizability; identification of unique contribution(s) to scholarship in a discipline or field	100-105
S19	Limitations	Trustworthiness and limitations of findings	102
		**OTHER**	
S20	Conflicts of interest	Potential sources of influence or perceived influence on study conduct and conclusions; how these were managed	105
S21	Funding	Sources of funding and other support; role of funders in data collection, interpretation, and reporting	105

*Reference: O’Brien BC, Harris IB, Beckman TJ, Reed DA, Cook DA. Standards for reporting qualitative research: a synthesis of recommendations. Academic Medicine. 2014. 89 (9). DOI:10.1097/ACM. 0000000000000388.

***Study aims***

The city of Dezful was chosen as the center of this study because the area is known to have a very high mortality rate of motorcyclists (known as ‘City of Motorcycles’)^6^ and in face-to-face visits to the police offices, local authorities wanted more research to be conducted on this area. Studies conducted in this city show that over 50% of those killed in 2015 were due to RTIs^5^ Given the above and the fact that almost all organizations acknowledged the problem of traffic accidents (especially for motorcyclists) in Dezful, this study aimed to identify environmental and legal factors affecting traffic accidents in the city and provide suggestions in line with solving them by all relevant stakeholders to gain a wide range of experiences. According to the conditions described above, the researchers intended to use the theories and experiences of those involved in this field, and following the previous work done in the city, which had looked into human factors in the second phase, the researchers examined two other factors affecting accidents, namely environmental and legal factors. For this purpose, it was necessary to survey and assess the needs of key and knowledgeable people in this field in order to access the necessary and comprehensive information and from different aspects.

The study was made operational after holding 5 focus group meetings for exchange of views. In the qualitative phase, focus group meetings with key informants were used to identify the legal and environmental factors impacting RTIs among motorcyclists and then in the quantitative part the key informants prioritized effective strategies in reducing number of RTIs among motorcyclists. The focus group meetings were all held at the venue of deputy of education at Dezful University of Medical Sciences (DUMS). They meetings were usually held every 15 to 30 days.

**Qualitative phase**

***Participants recruitment and eligibility criteria***

At first, a general framework for selecting key informants was designed and relevant criteria were identified (stated below). Then, referring to the traffic police department of the city and while stating the aims and consulting in this field, those organizations that were somehow involved in the issue of traffic accidents in Dezful city and in a way had experience in this field were identified and listed. Then, with the coordination of ’Traffic Police’ they agreed on a date for holding the first specific meeting and an invitation letter was prepared and signed by the "Traffic Police" commander addressed to the officials and experts of these organizations to participate in the first briefing session. Then, referring to the organizations in person, and stating the purpose of the invitation for the survey through holding meetings, the invitation was submitted to the relevant people.

The key informants of the study in Dezful were: Police Force Commander of the city, Traffic Police Chief, Head of Police Traffic Operations, Head of Traffic Education and Culture, Roads Traffic Police, DUMS (i.e. Vice Chancellor for Health, Vice Chancellor for Research, Head of Health Education Department, Vice Chancellor for Health, Expert of Non-Communicable Diseases (Accidents) of the Prevention Unit of Deputy of Health, Head of the Prevention Unit of Diseases of the Deputy of Health, Director of 115^1^ Emergency Medical Center), Administrator of Dezful General Hospital, Head of Emergency Department of Dezful General Hospital, Emergency Physician of Dezful General Hospital, Head of Education & Training Department, Governor, Mayor, Deputy of Municipal Affairs of Dezful municipality, Head of Urban Bus Organization, Head of Fire Department, Head of Road and Transportation Department, Head of Water Department, Head of Electricity Department, Head of Gas Department, Psychology professors of Dezful Branch of Islamic Azad University, and Dezful Islamic Council members. 

Inclusion criteria: The criteria for selecting key informants to attend the meetings were as follows:

1. Given the purpose of the research regarding the use of participants' experiences of events, people who had objectively and personally experienced this phenomenon (scientifically or practically) were included in this research. 

2. People from different departments that according to the data obtained from texts, reports and recommendations of Traffic Police and other relevant bodies, in their tasks' description there was some sort of relationship with the issue of traffic accidents.

3. The organizations or departments that were located in Dezful city.

4. People interested in participating in the meetings and had an idea in mind to solve the problem.

5. People who acknowledged that traffic accidents, especially accidents involving motorcyclists in the city of Dezful, and felt it was a serious issue and required intervention.

6. The person in charge of the organization or an expert introduced by the person in charge of that organization.

Exclusion criteria: Exclusion criteria also included the following:

1. Being reluctant to participate in the study;

2. Not attending the meetings regularly; and 

3. Not having scientific and practical understanding of traffic accidents.

**Guide questions:**

The guide questions in the focus group were:

1. What are the effective factors in traffic accidents?

2. Among the factors which one is related to environmental factors and which one is related to legal factors?

3. What strategies do you recommend to solve the environmental and legal factors affecting motorcycle RTIs?

***Data collection***

In order to collect data in the qualitative phase, formal focus group meetings were held until achieving data saturation. The five focus groups were held in Persian and were reviewed and modified by experts and participants. Appendix 2  shows the items dealt with in all the meetings held. The first two meetings were held to identify the legal and environmental factors impacting RTIs among motorcyclists (i.e. the qualitative phase). The third meeting was held to examine the proposed solutions by the key informants to be used in making the prioritization matrix. All the focus groups lasted between 2-3 hours.

**Appendix 2:** Summary of the first three focus groups meetings held for qualitative phase.

Meeting No.	Goal	Participants(Key informants)	Duration; Date	Measures	Approvals
1	Introducing the research & getting acquainted with concerned parties & paving the ground for attracting participation	18 people from 11 organizations in Dezful: 3 members from each of the following organizations: The Education & Training Department, traffic police, Dezful University of Medical Sciences (DUMS), 2 members from each of these: the Police forces, & the Municipality; 1 member each from each of the following ones: Urban Bus Organization, Gas Department, Road and Urban Planning Office, Fire department, & Electricity Department	3 hours; 30 May 2017	Describing objectives of the program, review of texts (Iran and the world), data obtained from the previous phase of the program (interviews with students, motorcyclists, Traffic Police personnel, education & training counseling center and the people of Dezful), the question raised was: "Given the description of the program, are you willing to cooperate and provide your experiences, comments and suggestions at no cost and voluntarily?”	Agreeing upon finding a solution to the problem by giving suggestions and solutions, invite other relevant organizations according to the snowball method to participate in the next meeting
2	Exchanging views on decision-making on required information	13 people from 10 organizations in Dezful: 2 members from the each of the following organizations: Iran Insurance Company, Dezful General Hospital; and 1 member from each of the following ones: Welfare Office, Urban Bus Organization, traffic police, DUMS, Telecommunication Office, Gas Department, Education & Training Department.	2 hours & 10 minutes; 15 June 2017	Visiting other proposed organizations in person and increasing the number of participants, introducing all the factors affecting traffic accidents (Table 3), highlighting two environmental and legal factors for participants and discussing them, determining the problems of the city according to these two factors, the question was: “What do you think can be added to this table?”	Consensus on the list of required information, review and search for possible solutions to reduce number of traffic accidents with respect to both environmental and legal factors
3	Proposing, discussing and exchanging views on ways to reduce RTIs according to legal and environmental factors	14 people from 11 organizations in Dezful: 2 members from each of the following organizations: 115 Medical Emergency, Police Department, Traffic Police, and DUMS; and 1 member from each of the following ones: Gas Department, Urban Bus Organization, Municipality, Iran Insurance Company, Dezful General Hospital, Education Training Department, & Municipality	2 hours; 29 June 2017	The questions in this session were: “1. Given your work experience in your organization, offer your proposed solution. 2. What do you think of the solutions offered by other participants?”Presenting suggested solutions by every single participant, recording solutions proposed by researchers, discussing and challenging solutions by all participants	Examining the proposed solutions for the next meeting, recording them in a decision making matrix for prioritization

The following principles were considered for holding all the focus group meetings and for attracting the participation of organizations:

1. Making efforts to attract maximum number of relevant organizations and diversity;

2. Observing order in holding meetings, providing accurate and timely information on each meeting;

3. Paying attention to the group dynamics during the meetings and encouraging the participation of all participants;

4. Training and providing information related to discussion; and decision making prior to the formal start of meetings;

5. Taking into account reaching consensus in the decision-making process in meetings;

6. Determining the expectations and defining the role of each organization;

7. Inviting Traffic Police staff officially;

8. Accepting the responsibility of chairing the meeting by Traffic Police;

and

9. Proposing solutions, as much as possible, in compliance with the aims of the project.

After taking into account the above points, the following measures were taken in all focus group meetings:

1. Initially, a telephone call was made with the head of the office to coordinate the meeting date- only in the first meeting; a written invitation was issued and delivered in person at the office, and holding the rest of the meetings were coordinated by phone.

2. After the phone call the day before the meeting, the time, date and venue of the meeting were reminded again via SMS, and participants were provided with the agenda.

3. Whereas the participants varied in each session, in order to keep the newcomers informed, at the beginning of each session, the aim of the session was presented through submitting a report summarizing the approvals of previous sessions and/or issues raised in them. 

4. The subjects that needed to be agreed upon were raised and the participants commented on them individually. 

5. Other retired experts who were familiar with the case were also present in the meetings. 

6. During the meetings, a voice recorder was used to record all the speeches (after obtaining the permission of the participants). Once the meeting finished, the guests recorded speeches were carefully listened to and the minutes were typed word by word. In the next step, the minute of the meeting was summarized and the important contents were categorized in a format. In each session, a printout of the minute of the meeting summary was provided to the participants for their information.

7. The draft for each session related to the topic was prepared for discussion, correction and finalization.

Sample selection continued until saturation. Participation included presenting suggestions, comments and experiences, and attending the meetings. All the personnel that were working as the head or expert of that body at the time of the present study constituted our study population. At each meeting, a question was raised, i.e. the ‘guide questions’ used in the focus group previously stated, and participants could freely talk about it and express their ideas. The audio comments presented in every single session were recorded and a summary of the main topics discussed in each session and its approvals was presented to each participant in the following session to ensure that the researchers have properly interpreted the participant's comments (a member check).^17^ The interviews were transcribed verbatim in Persian.

Decisions on the number of group discussion sessions were made by data saturation. During the sessions, whenever it was felt that the participants were not moving in the direction of the aims, facilitator’s efforts were made to lead them to the main topic by raising guiding topics. To get more information and making the issue transparent, questions were used such as: "Could you shed some more light on it?" Or "What do you mean by that?" Or "Can you guide us providing a practical example?".

In each session, the data collected using focus group discussions continued until no new topics in the data were presented by the participants, and the researchers were assured that data saturation had been achieved.^17,18^ Data saturation occurs when in focus group sessions, the comments presented do not indicate a new topic or introduce new elements of an existing topic.^6^

***Data analysis***

In the qualitative phase, all the focus group discussions, ideas and solutions mentioned in each session, which were recorded by voice recorder, were repeatedly listened to and transcribed verbatim and then analyzed according to the recommendations of Strauss and Corbin.^19,20^ For instance, data collection and analysis were performed simultaneously to identify ideas.^21^ They were then used to lead the next session. The main researcher and one of the colleagues studied the Persian texts carefully to get a general understanding of the full text. Afterward, the text of the sessions was compared with the audio files recorded for accuracy.

For each session, the lead researcher returned to the participants and briefly reviewed the content of the session (via providing the minutes and approvals of the previous session). The data collection process continued until each concept was saturated and until collection of other data could not provide the research team with new information.

During the focus group meetings, all the determined concepts were discussed up to saturation.^21^ In order to ensure the dependability and adequacy of the data, the supervision of expert colleagues was benefitted from and the content of the meetings was presented to the research colleagues and a qualitative research expert for a more detailed review of the coding. The content of the sessions was studied several times after implementation and resulted in general cognizance.^22^ In the coding stage, all the contents of the sessions were read several times and the key words or phrases in the text were taken into consideration. However, the points which had nothing to do with the research topic raised by participants were deleted. During this step, the initial codes were extracted. Codes and data were compared to detect any similarities and discrepancies, and then categories and subgroups were created.^21^

The initial codes were refined after each session and according to the consistency and homogeneity of each code, it was placed under a more general concept and this process was repeated many times until the initial codes that are more semantically similar to each other were each organized as sub-concepts, and all sub-concepts were also organized based on conceptual homogeneity. In order to clarify the categorization, and to eliminate the existing contradictions in the interpretation, the process of repeated return to the texts was done.^22^ The main message and the key meaning and concepts hidden in each of the phrases was then extracted and recorded. This was done for each paragraph and the full text of each session. In some of the sentences, the participants used terms that could be used directly as a code, and in some cases, there was a concept hidden in the sentences and the whole text, which according to the intention of the participant, a concept was attached to it, and was considered as a primary code.^22^ The lead researcher was careful not to be bound by predefined concepts and to allow additional codes of data to appear inductively. Following this trend, the principal researcher allowed broader themes to emerge, with more reflection on the code. In order to confirm the accuracy and interpretation of the data during the coding process, and to create the themes obtained from the findings, a discussion was conducted and agreement was reached among the research team members. Then, the subjects obtained were reviewed by a bilingual health professional to make them legible.^21^ In addition, Khorasani-Zavareh randomly selected more than 40% of the texts, and were read and coded separately by two study consultants who held regular meetings to discuss and resolve coding issues.^23^

Finally, the research team discussed the final phrases and contents. Based on this, a preliminary set of codes, sub-concepts and sub-concepts was created out of the focus group meetings and approved by the research team. At the end of the coding step, the main variables were identified. To this end, the research team (two PhD holders in Health Education and Health Promotion, one PhD holder in Healthcare Services Management and one person with MSc degree in Nursing) also agreed on the main variables that were raised and discussed for the final decision.

Triangulation method was used by the third researcher independently in reviewing the texts, followed by a group discussion to ensure the credibility of the results and the use of agreement for all topics,^19,24^ as well as evaluation criteria to establish the trustworthiness of qualitative data (i.e. credibility, dependability, confirmability, and transferability were also used.^23^

***The quantitative phase***

***Participants recruitment and eligibility criteria***

Inclusion criteria: The criteria for selecting key informants to attend the meetings in the quantitative phase were the same as the ones stated in the qualitative phase.

Exclusion criteria: The criteria for excluding key informants were as follows:

1. Those who were not present in the meetings or in the prioritization session.

2. If they did not completely fill the prioritization matrix form.

3. If they did not submit the scoring prioritization form at the end of the session.

***Data collection***

In order to collect the data in the quantitative phase, formal participatory group meetings were held. All informants who participated in the first to third sessions, in which all the data gathered as legal and environmental factors in the qualitative phase, were asked to prioritize the ones that were the most effective strategies to prevent RTIs among motorcyclist in Dezful in the fourth and fifth session (i.e. the quantitative phase). And then they were asked to give score to those solutions. More detail information about the fourth and fifth session is found in appendix 3.

**appendix 3:** Summary of the fourth and fifth focus groups meetings held for quantitative phase

Meeting No.	Goal	Participants(Key informants)	Duration; Date	Measures	Approvals
4	Prioritizing the proposed solutions	18 people from 14 organizations in Dezful: 2 members from each of the following organizations: Governor's Office, DUMS, 115 Medical Emergency, Dezful General Hospital, Traffic Police Department, & Iran Insurance company;1 member from each of the following ones: Fire Department, Gas Department, Education & Training Department, Municipality, City Council, & Urban Bus Organization	2 hours & 50 minutes; 27 July 2017	The question in this meeting was: "From your point of view and according to your experiences, how much priority and importance do each of the proposed solutions have, and are they applicable, changeable and cost effective?"Providing the participants with matrix forms to be filled in and prioritizing the proposed solutions with the priority matrix tool (Table 3) by the participants based on these criteria: priority, importance, changeability, cost- effectiveness, applicability, and result giving score from 1-5	Summing the scores by the researchers and announcing in the next session, selection of priorities and offering final solutions by the participants based on the obtained scores
5	Making final decision on strategies to reduce number of traffic accidents	18 people from 13 organizations in Dezful: 3 members from each of the following organizations: Traffic Police and DUMS; 2 members from Dezful Islamic Azad University; 1 member from each of the following ones: Urban Bus Organization, Fire Department, municipality, motorcycle riding training school, gas department, Friday Prayer Leaders' office, Iran insurance Company, Dezful Islamic Council, Sports and Youth Department, Dezful General Hospital	2 hours & 45 minutes; 10 August 2017	The final question was "What do you think of the solutions that have the highest score? Do you agree or disagree?,” Expressing, discussing and exchanging views on the determined priorities that were given the highest score according to the priority process at the previous session as a priority , as the main axes for subsequent interventions	Final consensus on priorities, informing and announcing them to participants and other stakeholders

The decision was made by consensus based on the available data, using decision matrices. The procedure was that the matrices were prepared, categorized and provided to the participants according to the suggestions received from the various above-mentioned sources, and they were asked to select each option based on the criteria of priority, importance, changeability, cost-effectiveness, applicability and result. And they were asked to score them from 1-5. Then the scores in all filled-in forms were calculated one by one and arranged in a table. Finally, the obtained scores were announced to the participants and at their discretion, a number of bids that received the highest score were selected.

In the quantitative phase, the same key informants who were chosen by the research team, in the qualitative phase were used in the research.

***Data analysis***

In the quantitative part, key informants were asked to prioritize all the strategies identified in the qualitative phase through a self-made scoring matrix (Table 1). Based on the suggestions received from qualitative phase, the matrices were prepared, categorized and provided to the key informants, and they were asked to select each option based on the criteria of priority, importance, changeability, cost-effectiveness, applicability, and results, and score them from 1 to 5. The scores in all the filled-in forms were calculated, and they were sorted in a table from highest to the lowest scores. Finally, the scores obtained were announced to the members, and at their discretion, a number of bids that received the highest scores were selected.

**Table 1:** Data collection form on road traffic injuries (RTIs) reduction strategies.

Matrix for prioritizing proposed solutions related to environmental and legal factors of reducing RTIs						
Proposed solutions/ strategy	Priority	Importance	Changeability	Cost-effectiveness	Applicability	Result
						
						
						
						
						
						
						
						

***Ethical considerations***

In order to consider the research ethics, the following measure were taken:

 Prior to collecting information from the key informants, the purpose of the research was expressed and they expressed their consent.

 Code of Ethics in Research of Dezful University of Medical Sciences (DUMS) in Iran approved the study protocol (Approval ID: IR.DUMS.REC.1395.15)

 All participants provided their written consent to participate in the study.

 Participants were advised that participation was voluntary, and they were free to leave the study at any time.

 Prior to the commencement of study, the key informants were briefed about the ethical principles and aims, the authority to withdraw from the research, obtaining permission to record the interview, and the authority to withdraw from answering some questions.

 Ethical considerations regarding the confidentiality of the names of the bidders were observed.

^1.^ **115** is a medical emergency call number in Iran.

## Results

***Qualitative results***

The participants (i.e. key informants) in the meetings held, were in the age group of 40-60 years and either held a bachelor's degree or a higher degree. In total, until reaching data saturation, which included 5 formal sessions, a total of 83 persons/session (some participants participated in all the five meetings in both the quantitative and qualitative phase) including the officials and experts of the relevant organizations of Dezful city attended the meeting for 12 hours and 45 minutes. Given the results obtained from the content of the meetings, finally, the research team achieved 45 basic codes in the legal factors field (appendix 4) and 8 basic codes in the field of environmental factors ( appendix 5).

**appendix 4:** Basic codes of legal factors

Making visible: obstacles, motorcycles and pedestrians with paints and stickers or glossy stickers
Further monitoring and training of riders’ license issuance schools
Seriousness in enforcing the laws and dealing legally and severely with violators
Continuous marking of roads and streets
Random check of motorcyclists riders’ license
Construction of public parking lots in crowded zones
Severity in driver’s license issuance
Issuing permission to ride motorcycles only at certain hours of the day and night and not allowing them to ride motorcycles during busy and hazardous hours
In the event of an accident, the insurance company will pay indemnity just in case the guilty motorcyclist has attended a mandatory 20-hour educational course, otherwise the insurance contract will be terminated
Completion of the violation registration system in the Dispute Resolution Council and escalation of fines
Not allowing people under 18 years of age to ride motorcycles
Setting up a technical examination center for motorcycles
In case motorcyclists over 18 violate the regulations, they should be heavily fined
Reducing the number of motorcycles in the city applying odd and even plate numbers' plan
Announcements by the municipality and traffic police on reforms made all over the city and providing them with necessary guidance
Making articles 11 and 20 (law of traffic violations investigations) transparent, and enforcing them severely
Installation of speed bumps and barriers on sidewalks to prevent motorcyclists riding on the sidewalks
Implementing incentive plans to issue driver's license cheaper than the approved price for one week for the target age group
Encourage cycling in schools, and close main streets to motorcyclists and just allow bike riders to ride bikes
Raising fine rates
If someone does not hold a driver's license and proves guilty in an accident, he himself is to bear the treatment expenditure
Severely enforcing the law for people who illegally let people ride their motorcycles
Making psychological testing mandatory for those who violate regulations
Rising fuel prices for offending motorcyclists
Implementing identical procedure in dealing with offending motorcyclists
Necessity of holding military service completion certificate and driver's license when purchasing a motorcycle or changing license plates
Making coordination with the manufacturer to reduce the speed of ordinary motorcycles by installing a part for riding in the city and allowing other fast motorcycles to be ridden on the race tracks
Prosecuting parents who let their children under legal age to ride their motorcycles
Increasing traffic light waiting time
Increasing the number of police forces
Providing students with free bikes or bikes with installment to institutionalize cycling and encourage them to cycle
Making the process of obtaining driver's license longer via holding mandatory and psychology classes
Making theoretical testing of regulations easier
Issuing driver's license for people of 16 years of age and above

**appendix 5:** Basic codes of environmental factors

Identifying the places where traffic signs are covered by trees, and reporting through the 137 call center
Identifying and reporting shoulderless and hazardous roads by municipality
Identifying and reporting accident-causing potholes through the 137 call center
Prohibiting traffic while it is dusty
Closing down recreation centers and shops at midnight and twilight time
Cleaning and washing streets and roads by municipality, especially in fall and winter
Building a separate route for motorcyclists and not allowing them to ride in any other routes
Checking vehicles problems for cold and rainy days

In each of the two main themes (i.e. legal factors and environmental factors), sample quotations of suggestions made by the participants in the focus group meetings of the 6 main concepts in the legal factors field and 3 main concepts in the environmental factors field are shown. The following are some of the concepts and quotations of suggestions made by the participants in the meetings.

**First Theme: Legal factors**

***1. Making visible: obstacles, motorcycles and pedestrians with paints and stickers or glossy stickers***

The participants in the meeting discussed visibility and being visible and pointed out that most of the motorcyclists in Dezful had accidents at night when it is dark, and motorcyclists are not pretty visible; therefore, it causes accidents; for instance:

"Most of the accidents that happened at night originate from factors such as dark clothes worn by motorcyclists, lack of lighting in the roads, the failure to replace the broken down lamps of motorcycles, which are unfortunately not observed.” (Police in charge of traffic training)

Participants also noted that this could be prevented by establishing a culture on the part of pedestrians and motorcyclists to wear light color clothes and it could be also achieved using ways to enhance drivers' sight, and the municipality should be more seriously pursue the issue of street lighting. Some participants referred to the problems created by local organizations such as the Water, Gas and Electricity departments and believed they were responsible for the accidents; for instance:

*"These departments have to make drilling in the city to fulfill their tasks, but the delay in filling in these trenches and lack of using proper warning signs in places where project is going on may cause injuries ...” (Representative of the Fire Department)*

***2. Further monitoring and training of riders’ license issuance schools***

The meetings were attended by two heads of motorcycle riding training schools issuing motorcycle riders' license working under the supervision of the traffic police. While referring to the available data from the previous phase of the program and their experiences, some city officials, were of the opinion that the training process in the training schools was not taken seriously and declared the necessity of more supervision in this area; For instance:

"Many senior students of high school who have attended the trainings at the relevant schools to get a rider's license say, it is not taken seriously, and as far as you make the payment, without any training, they just sign the form.” (Health Expert at Education & Training Department)

Given the importance of training and acquiring the necessary skills for motorcycling, the participants reminded the necessity of taking the issue seriously and monitoring it.

***3. Seriousness in enforcing the laws and dealing legally and seriously with violators***

Almost all participants were of the opinion that high-risk behaviors such as; young people performing dramatic movements, zigzagging, lane splitting, and not observing traffic rules, while it is noteworthy that unfortunately, other members of the society have accidents due to other reasons: in some cases a few wands of families ride on a motorcycle; in some other cases ladies wearing long dresses (e.g. Chadors) do not handle it properly and it is vacuumed inside the motorcycle wheels and leads to falling of all riders. Another case is not replacing the broken down lights of motorcycles due to laziness and not being visible in dark places at night which lead to accidents, and in all these cases, the traffic police officers present in the passages simply ignore those motorcyclists who disregard the laws; For instance

*"Police officers in places where there are red light signs and when the light is red, motorcyclists pass by and do not pay attention to the red light and the police do not react and allow motorcyclists to be fined or even stopped without being fined. Cross the road …” (Accident prevention expert of DUMS)*

The traffic police pointed out that in some cases, due to the lack of police officers and the lack of permission to chase motorcyclists, practically no measure could be taken, because chasing a motorcycle may endanger the motorcyclists or other road users' life. Also, the law does not support police officers. For instance:

*"Last week, we confiscated the motorcycle of a young offending motorcyclist and took him to the parking lot. On the way to the parking lot, we were told to return the motorcycle to its owner because this person is a son of influential person ...” (A traffic operations expert)*

The economic and livelihood problems of the people were discussed and it was said that the reason for the drivers' violations could be more related to these problems. Such as not having a technical inspection, motorcycle defects, not insuring the vehicle, working in old age and using the vehicle, etc.

***4. Continuous marking of roads and streets***

Participants discussed poor performance in displaying traffic signs and noted the importance of these lines in guiding drivers and helping to comply with traffic rules. For example:

*"Marking are good guides for drivers. On my way from home to university, I have noticed that the color of the marking is constantly erased, each color does not last more than a month.” (Faculty member of the Dezful Branch of Islamic Azad University)*

The traffic police chief, while acknowledging the importance of markings in observing the rules by drivers and preventing violations, said that the paint used for this purpose was of poor quality; they said:

"Dezful Municipality spends a lot of money on doing these markings. The road marking work is done in cooperation with us and the municipality, but the paints that are used are not suitable for this purpose, and once vehicles pass by and due to sun and rain are easily faded." (Traffic Police chief)

***5. Random check of motorcyclists’ license***

In this regard, the participants said that the traffic police never check the motorcyclists' riders' license unless there is an accident. It seems that the strategy of randomly checking their license may urge those who do not hold riders' license to take necessary action in this regard; especially for those who are below the legal age and ride around the city as a rider; there is a need for serious pursuit. For instance:

"We advertise a lot and urge people to refer to us to be trained and to get riders' license to present to the police whenever necessary, but in practice the number of people who refer to us is small because they start riding motorcycles at an early age and no one checks their riders' license.” (Head of riding training school issuing motorcycle riders' license)

Meanwhile, participants also asked the traffic police chief about the number of licenses issued in the city, and it came out that there is no comprehensive and accurate statistics on the issue: 

*"In case an accident leads to injury and requires a sketch of the accident site, the riders' license is checked and registered. If it leads to death, it is recorded by a forensic pathologist. Only in these two cases we know the number of riders' licenses …” (Traffic Police operations expert)*

***6- Construction of public parking lots in crowded zones***

This sub-theme could be classified in both legal and environmental theme and it has an overlap. But because the traffic police of each city in Iran is responsible for giving the permission for the construction of parking lots, it has been classified under legal one.

The urban structure of Dezful city, especially in the main and central areas of the city, is old and narrow and due to the accumulation of businesses, clinics, physicians' offices, laboratories, etc., it is so crowded. All the participants, while acknowledging this issue the necessity of facilitating people's affairs, considered the construction of suitable parking lots in these areas as one of the best solutions to solve the problem. For instance:

*“Many people who refer to this zone to refer to pathology laboratories, cannot find parking space; so they park dubbing or block the entrances of the side streets leading to these avenues and cause disturbances, quarrel and accidents, which in turn exacerbates the congestion.” (Vice Chancellor for Health, DUMS)*

Of course, some suggestions were made in this regard, which are applicable in new zones of the city, but are less applicable in the central and downtown of the city:

*"It is necessary to remedy the problem in these zones; in the case of an accident in these zones, the relief task becomes difficult and the when the ambulance wants to enter the zone faces a lot of problems. Separate routes must be created for this purpose." (115 Emergency Medical Expert)*

As far as all participants knew that it was impossible to make changes in these crowded zones of the city and expand them, the only way out is to create parking lots in these zones. It was also noted that lack of parking lots in these zones will increase citizens' use of motorcycles. 

**Second theme: Environmental factors**

***1. Identifying places where traffic signs are covered with trees, and reporting through the 137 call center^2^***

Regarding the visibility of traffic signs, the participants reached agreement that provided that municipality and traffic police do further review on the condition of passages and streets, many accidents could have been prevented in the city. In many cases the growth of trees and neglecting it, has led to the coverage of signboards or exerting pressure on power lines and has led to accidents; for instance:

*"Some of the deaths emanated from this issue are simple and preventable. Motorcyclists collide with vehicles, in particular due to cases that traffic signs on the main streets of the city are not visible. Of course, this may not be a problem for local drivers but it will be problematic for other drivers or inexperienced young people…” (Forensic expert)*

Participants also exchanged views on other environmental issues, for instance:

*"Sometimes vehicles are parked in places that create problems for other drivers, so that they cannot see everything; and unfortunately because they live there, they do this over and over again. The interesting point is that when lack of visibility of the drivers causes an accident, immediately they move their vehicles.” (Traffic Police chief)*

With regard to solving this problem, which requires providing a lot of information from the point of view of other participants, the municipal traffic expert said that in case citizens notice any problems such trees in the middle of the boulevards not being pruned, the broken branches of the trees, any obstacles along the streets such as building materials, motorcycles or cars parked in inappropriate places, they can call 137 to follow up and solve the problem.

***2. Identifying and reporting shoulderless and hazardous roads by municipality***

With regard to suburban roads and villages around the city the participants pointed out that tragic accidents and deaths always occur in these roads. As these roads are not main ones, they have not been authorities' center of attention, while the city is the hub of agriculture, and these roads are widely used for the people's economic activities, for instance:

*" There are many problems with accidents on rural roads in the suburbs of Dezful. The road is not in suitable conditions, it has no shoulders, it is not paved. They need a lot of follow-up for the transportation of agricultural products and residents moving from one place to another.” (Traffic Police Chief)*

From the participants' point of view, just as urban streets are important and should be considered, people in rural and suburban areas also face traffic problems that threaten their lives, for instance:

*"In the Sharak-e-Hamzeh village "near Dezful", in particular, there are a lot of problems. We have also installed banners in this area to make people in the area aware, and drive at a lower speed ... The road infrastructure needs a lot of credit to be improved ...” (Expert of Road and Urban Development Department)*

***3. Identifying and reporting accident-causing potholes through the 137 call center***

In this context, the participants had previously discussed implementation of traffic laws, and referred to the chronic problem of the city: poor conditions of urban roads, the existence of numerous potholes and bumps created by various organizations or the citizens, and non-standard obstacles created by the municipality to reduce the drivers' speed. The participants stated that we acknowledge that the asphalt will be damaged due to the hot weather of the city, the colors of the obstacles will fade due to intense sunlight, but this issue should be further followed up; for instance:

*"Every day we witness a new hole in the streets, the suspension system of the cars are damaged due to these holes, and patients in critical situation have to bear with these holes and obstacles and bumps until they reach the hospital; of course if they reach hospital safe and sound.” (Dezful Grand Hospital administrator)*

Participants also complained about the creation of handmade barriers by traffic police and the municipality, for instance:

*"I wonder whether these officials themselves drive on these streets? Don't they notice these problems? At least they are expected not to create additional obstacles.” (Director of the Non-Communicable Diseases Prevention Department (*

In this case, the municipal expert spoke about the municipal information system and offered his proposed solution to solve this problem of through informing the 137 call center.

***Quantitative results***

**The legal factors**

After summarizing the comments and suggestions of the key informants, during the fourth and fifth session of the focus group meetings, the prioritization of legal solutions was finalized. By using priority matrix in the quantitative phase, the key informants finally noticed 6 main concepts in the legal factors field and 3 main concepts in the environmental factors field as the most effective strategies in preventing motorcycle RTIs.

The results of the scoring process obtained from key informants in the focus group meetings to reduce motorcycle RTIs for the main legal factors and the total scores using the decision matrix are provided in Table 2.The first six had the highest scores and were prioritized as the most effective strategies to reduce motorcycle RTIs.

**Table 2:** Matrix for prioritizing proposed solutions related to legal factors of reducing RTIs.

Related factor and proposed solutions	Total score*
Making visible: obstacles, motorcycles and pedestrians with paints and stickers or glossy stickers	559
Further monitoring and training of riders’ license issuance schools	552
Seriousness in enforcing the laws and dealing legally and severely with violators	549
Continuous marking of roads and streets	541
Random check of motorcyclists riders’ license	528
Construction of public parking lots in crowded zones	506
Severity in driver’s license issuance	499
Issuing permission to ride motorcycles only at certain hours of the day and night and not allowing them to ride motorcycles during busy and hazardous hours	498
In the event of an accident, the insurance company will pay indemnity just in case the guilty motorcyclist has attended a mandatory 20-hour educational course, otherwise the insurance contract will be terminated	497
Completion of the violation registration system in the Dispute Resolution Council and escalation of fines	492
Not allowing people under 18 years of age to ride motorcycles	488
Setting up a technical examination center for motorcycles	473
In case motorcyclists over 18 violate the regulations, they should be heavily fined	470
Reducing the number of motorcycles in the city applying odd and even plate numbers' plan	457
Announcements by the municipality and traffic police on reforms made all over the city and providing them with necessary guidance	454
Making articles 11 and 20 (law of traffic violations investigations) transparent, and enforcing them severely	449
Installation of speed bumps and barriers on sidewalks to prevent motorcyclists riding on the sidewalks	448
Implementing incentive plans to issue driver's license cheaper than the approved price for one week for the target age group	444
Encourage cycling in schools, and close main streets to motorcyclists and just allow bike riders to ride bikes	442
Raising fine rates	435
If someone does not hold a driver's license and proves guilty in an accident, he himself is to bear the treatment expenditure	427
Severely enforcing the law for people who illegally let people ride their motorcycles	421
Making psychological testing mandatory for those who violate regulations	411
Rising fuel prices for offending motorcyclists	398
Implementing identical procedure in dealing with offending motorcyclists	396
Necessity of holding military service completion certificate and driver's license when purchasing a motorcycle or changing license plates	380
Making coordination with the manufacturer to reduce the speed of ordinary motorcycles by installing a part for riding in the city and allowing other fast motorcycles to be ridden on the race tracks	380
Prosecuting parents who let their children under legal age to ride their motorcycles	375
Increasing traffic light waiting time	374
Increasing the number of police forces	357
Providing students with free bikes or bikes with installment to institutionalize cycling and encourage them to cycle	331
Making the process of obtaining driver's license longer via holding mandatory and psychology classes	318
Making theoretical testing of regulations easier	300
Issuing driver's license for people of 16 years of age and above	300

* The first six had the highest scores.

**The environmental factors**

After summarizing the comments and suggestions of the key informants, during the fourth and fifth session of the focus group meetings, the prioritization of environmental solutions was finalized.

The results of the scoring process obtained from key informants in the focus group meetings to reduce motorcycle RTIs for the main environmental factors and the total scores using the decision matrix are provided in Table 3. The first three had the highest scores and were prioritized as the most effective strategies to reduce motorcycle RTIs.

**Table 3:** Matrix for prioritizing proposed solutions related to environmental factors of reducing RTIs.

Related factor and proposed solutions	Total score*
Identifying the places where traffic signs are covered by trees, and reporting through the 137 call center*	545
Identifying and reporting shoulderless and hazardous roads by municipality	541
Identifying and reporting accident-causing potholes through the 137 call center	528
Prohibiting traffic while it is dusty	395
Closing down recreation centers and shops at midnight and twilight time	362
Cleaning and washing streets and roads by municipality, especially in fall and winter	354
Building a separate route for motorcyclists and not allowing them to ride in any other routes	329
Checking vehicles problems for cold and rainy days	301

* The first three had the highest scores.

Finally, at the end of the meetings, the participants stated that in addition to environmental and legal factors, human factors are always prominent and should be taken into account; and no distinction can be made between environmental, legal and human factors. It was also mentioned that some factors are not only for motorcycles but also for other vehicles, as well.

## Discussion

Numerous studies have dealt with statistical analysis of road accident factors. In the present study, key informants suggested strategies to reduce motorcycle RTIs focusing on legal and environmental factors.

The obtained data suggest that glossy labels need be used for better visibility. This suggestion had the highest score. Improper visibility is a key risk factor that threatens car passengers and pedestrians. Seeing and being seen is a basic safety prerequisite for all road users.^25^ Accordingly, and especially in low and middle income countries, not being seen well is one of the important factors in the occurrence of RTIs, especially for vulnerable road users, including pedestrians, cyclists and motorcyclists. This issue is of such importance that in 2004, WHO emphasized it as one of the five main strategies for preventing deaths due to RTIs.^26^ Insufficient visibility plays a key role in three types of accidents:^25^

(1) at nights- when vehicles collide with other vehicles moving at a slow speed, vehicles parked or pedestrians;

(2) during day time- when there is a side or rear-end collision; 

(3) at all times- when rear-end collision occurs in foggy situations. 

In most high income countries, front and rear lights and reflectors are essential for bicycle wheels. According to a research conducted in the Netherlands, 30% of bicycle crashes occur at night or early in the morning, which can be prevented by using bicycle lights. The use of clothing or tools or colored parts may make bicycle, pedestrians, passengers, and non-motorized vehicles more visible. Reflective vests also provide better visibility, but their cost and inadequacy in hot weather make them unsuitable for use in low and middle income countries.^25^

Unfortunately, in the city of Dezful, mostly the reduction of night vision, and vision problems among some drivers and pedestrians, as well as ignoring the necessity of changing out of order lamps of vehicles may lead to accidents in the darkness of night. After a while, due to passage of time, the white colored marking of roads and streets used in the city become almost invisible at night. The pedestrians wearing dark and walking on roads that do not have enough lighting, may also lead to vehicles colliding with pedestrians, and the driver might not react quickly when approaching and being seen. It has been reported that in case there is no proper lighting on the street, accidents caused due to not being seen are more dangerous for drivers.^27^

According to the study of Anarkooli et al., which is consistent with the findings of the present study, the probability of injury in single-passenger vehicle accidents such as motorcycles and bicycles in inadequate lighting conditions increases by 34%, while conversely, the probability of minor injuries decreases by 37%.^28^ According to people involved in this study, if motorcyclists who have to ride on dark roads, use visible color labels on their motorcycles, it may reduce rate of road traffic injuries; provided that is notified to them as a binding rule. 

Injuries caused by road traffic accidents affect all social groups in the society. WHO has recommended five points to the general public: 

• Do not drive fast

• Wear seat belts

• Use helmets 

• Do not drink alcohol while driving and

• Be visible on the road.^26^

Visibility or being seen on the road is a basic requirement for the safety of all those who use roads, especially vulnerable users. Inadequate visibility plays an important role during both day and night for road users. But this issue seems to be neglected now and has become a big challenge. This is true in low and middle-income countries such as Iran, where for cultural reasons, people wear dark clothes and find it unusual to use light clothes or reflectors. Legislation in this area is weak and reflectors are not available to the public.^26-29^

The results of other studies also indicate that driving in streets with poor lighting conditions, accounts for a large proportion of severe accidents.^30^ In Iran, to improve visibility, there is a need for extensive inter-organizational cooperation to produce reflectors on one hand, and make them available to the public, and to expand their use through campaigns, and on the other hand, the textile, auto, motorcycle and bicycles industries should try to use it in their products.^26^

Installation of informative and warning signs to make drivers more familiar with the rules and safety tips on the streets, severity in enforcing the laws and dealing decisively with violators and observing legal issues such as holding drivers' license, observing the priority and traffic regulations are also among priority issues the concerned people referred to. Failure to wear a seat belt is considered as one of the most important factors impacting the severity of vehicle collision injuries.^31^ In the Yau study, when passengers wore seat belts properly and when seat belts were not worn severe mortality rates were 24% and 45%, respectively.^23^ Not wearing a seat belt enhances the risk of injury and death by 24% by 80%, respectively.^32^

At a similar study conducted by Khorasani-Zavareh et al., it came out that while helmets and seat belts are compulsory in Iran, most motorcyclists do not wear helmets. It seems that they do not believe in it, and some of them even do not hold riders' license. In addition, improper driving is a problem in our country, and most drivers while disregarding safe behavior like to drive recklessly. It seems that lack of knowledge and trust in traffic law has led to underdeveloped traffic safety culture.^21^ Zamani-Alvijeh and coworkers’ study have mentioned that motorcyclists did not wear helmets.^23^ In another study in Iran mentioned that the reasons behind this are attributed to various cases: people living in the suburbs not being familiar with traffic laws, laziness in sticking to law in some cases, just relying on giving tickets to drivers and insufficient public cooperation with traffic police.^21^

A meta-analysis conducted in 2016 indicated that in most communities, reforming the law and enforcing it can be effective in reducing traffic accident rate.^30^ Also, a study conducted by Nazif-Munoz et al. in 2014 showed that reforming the traffic regulations could contribute to reduce the rate of pedestrians' death by 7%. Meanwhile, the severity of the police in enforcing the law leads to reduction in alcohol consumption, driving casualties by 18% in drivers, 8% in passengers, 60% in pedestrians and 60% in all deaths.^33^

In a study conducted by Khorasani-Zavareh and coworkers, all participants felt that this problem (i.e. RTIs) could be resolved by strengthening and enforcing the law, and providing traffic police with modern equipment for surveillance, they also believed that the law should delegate more authority to the police.^21^ In , Zamani-Alvijeh and coworkers’ study it was pointed out that the law was not properly enforced by the police, rather it was enforced randomly, i.e. not all violators were punished.^25^

One of the prioritized environmental factors was identifying and announcing accident-prone locations in terms of environmental conditions on the city's roads and streets. These environmental factors may reduce the visibility and timely reaction of drivers when crossing these areas; therefore, it is these areas should be identified and resolved by the relevant authorities. One of these factors is the road route or related environmental factors. In various studies, road surface and design have been proposed as important factors in reducing accidents, and it has been indicated that larger vehicles are exposed to more environmental hazards, and motorcyclists are more damaged in collisions, and to this end, solutions such as: separating the bikes and motorcycles path, roads with soft shoulder, and reducing the speed of the motorcycle have led to the reduction of the rate of accidents.^34-36^

In Abbasi and coworkers’ study, which was consistent with the issue, also reported that the lack of soft shoulder and their low width are among the most important road problems.^34^ In a review study, it came out that the issue of road safety has played an important role in many problems and high costs in traffic accidents. Road safety has also had a great impact on people's welfare, economy and productivity. A study in Kenya also revealed that rainy weather, especially at night, was a risk factor and made road users vulnerable. The use of new technologies such as facilities that absorb excess energy, improving visibility on motorcycles using advanced light bulbs and lights are recommended.^37^

According to all participants in the Khorasani-Zavareh and coworkers’ study, a large number of problems in the transportation system and its safety had something to do with one-way streets, insufficient road lighting, lack of special lanes for low-speed vehicles, lack of road shoulder, lack of adequate traffic signs on the roads, poor maintenance of roads and roads' blind spots. ^21^

In a study in Iran it also stressed the importance of human factors in the first place, as well as environmental and legal factors. According to WHO, monitoring these factors is related to full-scale intra-organizational and inter-organizational cooperation. It has also insisted on the necessity of paying attention to blind spots on roads, severe punishment for high-risk behaviors of drivers, installation of signs and behavior and attitude modification.^21^

***Limitations and strengths***

This study has inherent limitation of any qualitative research- the findings are limited by design and not generalizable to the larger motorcyclist population. Other limitations of the study included the following:

• the context of the city of Dezful may not be exactly the same of other cities in Iran. Therefore, we cannot generalize to all Iranian cities or even around the world;

• other governmental and private organizations may have more information about RTIs which were not invited or were not recognized to be invited or were not accessible to participate as a member of the focus groups; and

Despite the limitations, our method has followed essential aspects of a qualitative analysis:^38^

• the inclusion criteria were relevant to the research question; 

• representatives of most the organizations directly involved in RTIs in the city of Dezful participated in the focus groups;

• the data collection methods were appropriate for the specific aim of the study; 

• the data collection processes were rigorous and comprehensive to support saturation and robust descriptions of the information collected; 

• data was analyzed appropriately and results were corroborated by using multiple reviewers to ensure that participants' viewpoints were adequately interpreted and coded.

• typically, the number of participants in qualitative studies is relatively small; however, all participants were experienced and knowledgeable, and saturation of all concepts was achieved. Through using comparative analysis which means returning to data to validate and further develop categories, the data even proved this.

Furthermore, our results offer some insights into research that is not well established, and therefore, can be built upon in further studies. 

## Conclusion

The study aimed to reduce RTIs by reflecting on the legal and environmental factors, and prioritized strategies for promoting road traffic safety in Dezful. It is costly and not possible to successfully resolve legal and environmental factors with the budget allocated to one project. Therefore, making proposals operational requires the cooperation of organizations.

The present study could be of great use for stakeholders, policy makers, executives of educational organizations, law enforcement and urban architecture, and other organizations involved in RTIs and road accident prevention in Iran and cities similar to the ancient city of Dezful. These recommendations are divided into several categories proportionate with the dimensions of the research: Education & awareness, Enforcement and Legal Actions, Environmental Actions, Collaborations, Partnerships, and Lobbying, and Research; which is called 3ECR for short. These recommendations are available in Table 4.

**Table 4:** Recommendations for policy makers, practice and research to reduce motorcycle RTIs.

**EDUCATION & AWARENESS**
• More regular and consistent educational efforts focusing on groups, populations and areas that are prone to RTIs
• Establishment of a social network or online Management Information System (MIS) for "recording the experiences of injured or dead motorcyclists" by motorcyclists or their families to educate and inform the community and policymakers to use the data of these social networks and MIS to offer intervention solutions
• Creating an allocated page at Dezful University of Medical Sciences (DUMS) to provide information on the prevention of RTIs
• Regular education and training of government officials and entities involved in the prevention of RTIs
• Implementing road safety education through planning school syllabus especially for male junior and senior high schools students who are motorcyclists-to-be
• Public education to prevent the dangers threatening pedestrians, especially urging them not to wear dark clothes at night
• Use of new media and social networks to educate the public and provide them with information on road accidents' prevention
• Development of standards for producing effective educational media such as posters, infographics, infomotions, clips, videos, educational messages, etc. and notifying all organizations producing educational media in the field of RTIs
• Supervising the proper implementation of developing educational media such as posters, infographics, infomotions, clips, videos, educational messages, etc. and notifying all organizations involved in producing educational media in the field of RTIs and encouraging organizations that have produced works in this field; through media awards, etc. and through cooperation with all organizations involved in the issue
**LAW ENFORCEMENT AND LEGAL ACTIONS**
• Annual and random monthly technical inspection of motorcycles
• Civilian oversight of police negligence towards motorcyclists by taking photos and recording videos and sending it to the 197 police call center
• Legally dealing with motorcyclists under legal age, confiscation of their motorcycles and making the motorcyclists and their parents pledge not to do so again
• Declaring wearing helmets by motorcyclists and their companions mandatory
• Declaring wearing helmets for motorcyclists working with state institutions and service organizations, and taking disciplinary actions against such employees who violate the law
• Identifying accident-prone areas and further tightening the enforcement of traffic laws in these areas
• Strict implementation of laws and penalties related to driving violations through creating the required platform and legal support
• The seriousness of the authorities in enforcing and observing the existing laws by all drivers and motorcyclists
**ENVIRONMENTAL ACTIONS**
**A. Road Engineering and urban planning**
• Providing proper lighting for dark passages by municipalities
• Quick filling in of trenches dug by gas, water, electricity, telephone companies and those dug by other municipal services providers, and installing proper warning signs for pedestrians and motorcyclists
• Regular marking of streets and roads and using high quality and long-lasting paints
• Pruning trees in places where traffic signs and signs have been obscured
• People reporting the trenches which have not been filled in, traffic signs covered with tree branches, and other urban traffic barriers to the 137 municipal call center
• Improving road conditions: creating proper shoulder, repairing damaged parts of the asphalt and/or asphalting those parts without asphalt
• Practical use of suggestions made for policy makers, and knowledge-based and research-based decision-making by managers involved in RTIs
• Constant review and monitoring of probable environmental problems and those related to traffic signs and road signs that we face with on a daily basis on urban and suburban roads by the Municipality and the Department of Roads and Urban Development and Traffic Police department
**B. Preparing Parking lots**
• Constructing more parking lots near clinics, hospitals, markets, bazars, medical laboratories, and administrative complexes and old ancient sections of the city and facilitating laws for building parking lots by the private sector
• Ensuring good quality roads and streets are built in newly built sectors of the city with adequate width which are multi-lane
**COLLABORATIONS AND PARTNERSHIPS**
• Expanding and maintaining inter-organizational and intra-organizational communication between public and private organizations effective in preventing road accidents
• Inter-organizational coordination between organizations involved in providing services and urban development
• Lobbying to attract the attention of organizations that are effective in reducing and preventing RTIs rate
**RESEARCH**
• Conducting field research and in-depth interviews with motorcyclists and investigating the reasons why they do not observe motorcycling safety, especially the case of not wearing a helmet and not obtaining a motorcycle riders' license
• Conducting field research and in-depth interviews with motorcyclists and investigating the reasons why they do not observe motorcycling safety, especially not wearing a helmet and not obtaining a motorcycle riders' license in other cities with high statistics of motorcyclists' road accidents
• Conducting field research and in-depth interviews with motorcyclists who observe the safety of motorcycling, especially wearing helmets, and holding motorcycles rider's license to find elements that promote their safety behavior
• Publishing the executive report on researches conducted on road accidents prevention for the city executive managers
• Publishing the results of researches conducted on road accidents prevention in simple words and using new media facilities for the general public
• Investigating other factors impacting RTIs such as managerial-organizational ones or vehicle-related factors
• Conducting intervention studies aimed at determining the effectiveness of the proposed solutions implemented for the prevention of RTIs
• Given the cultural discrepancies and various context of each region that may affect the perception of others, to determine the impact of the proposed interventions and existing policies for prevention taking into account the infrastructure of each region

The first recommendations focus on education & awareness. Numerous organizations in small towns like Dezful may implement these recommendations in this sector: Education & Training Organization, local Islamic Republic of Iran Broadcasting (IRIB) offices, newspapers and other educational media, private sector educational institutions, traffic education institutions, Dezful University of Medical Sciences and its relevant educational departments, implementation of small educational projects by various university students among students and other target groups, advertising organizations, ....

The second recommendations concern Enforcement and Legal Actions. The organizations that are most involved in this field are traffic police, motorcycle manufacturers, and public organizations monitoring traffic police performance (e.g. 197)^1^

There are two types of executive recommendations for Environmental Actions: road engineering and urban planning and preparing parking lots. Municipalities and urban transportation organizations in the city and road transportation organizations are responsible for the main activities of the first group.

Perhaps one of the most important problems in cities is that organizations involved in the issue do not cooperate with each other and their duplicated actions to prevent RTIs is seen in their organizational mission. Therefore, collaboration and cooperation between the organizations involved in RTIs prevent duplication and waste of manpower, energy, budget, design and implementation, and leads to synergy in terms of facilities.

The last executive recommendations are related to organizations engaged in research in the field of RTIs: Dezful University of Medical Sciences, Education & Training Organization, Islamic Republic of Iran’s Police Forces (short form in Farsi NAJA) Research Group in Khuzestan Province and Dezful City, Urban and Intercity Transportation Organizations, Students' Research done by medical Sciences, Transportation Sciences, Behavioral Sciences, Social Sciences, etc.

**Abbreviations**

RTIs: Road Traffic Incidents; DUMS: Dezful University of Medical Sciences; DWI: driving while being intoxicated; IRIB: Islamic Republic of Iran Broadcasting; MIS: Management Information System

^2.^**137 call center**

^3. ^**197** is a call number in the police department which is used by the public as a civilian oversight for criticizing police performance and/or acknowledging good police conducts. 

**Acknowledgments** is a telephone-based communication system set up by municipality of most cities in Iran. Citizens may contact the municipality authorities by dialing 137 and offer their suggestions, raise their complaints or report any deficien-cy and request urban services. 

We would like to sincerely express our appreciation to the esteemed officials of various departments of Dezful city who participated in all the joint meetings aimed at reducing traffic accidents without any expectations, as well as the esteemed dean of Dezful University of Medical Sciences and officials of its various departments who accompanied the researchers in meetings and funded the project.
